# Clinical outcomes in patients with chronic lymphocytic leukemia with disease progression on ibrutinib

**DOI:** 10.1038/s41408-022-00721-6

**Published:** 2022-09-01

**Authors:** Paul J. Hampel, Kari G. Rabe, Timothy G. Call, Wei Ding, Jose F. Leis, Asher A. Chanan-Khan, Saad S. Kenderian, Eli Muchtar, Yucai Wang, Sikander Ailawadhi, Amber B. Koehler, Ricardo Parrondo, Susan M. Schwager, Taimur Sher, Curtis A. Hanson, Min Shi, Daniel L. Van Dyke, Esteban Braggio, Susan L. Slager, Neil E. Kay, Sameer A. Parikh

**Affiliations:** 1 Division of Hematology, Department of Medicine, Mayo Clinic, Rochester, MN USA; 2 Department of Quantitative Health Sciences, Mayo Clinic, Rochester, MN USA; 3 Division of Hematology and Medical Oncology, Mayo Clinic, Phoenix, AZ USA; 4 Division of Hematology and Medical Oncology, Mayo Clinic, Jacksonville, FL USA; 5 Department of Laboratory Medicine and Pathology, Mayo Clinic, Rochester, MN USA; 6 Department of Immunology, Mayo Clinic, Rochester, MN USA

**Keywords:** Chronic lymphocytic leukaemia, Cancer therapy

## Abstract

Patients with chronic lymphocytic leukemia (CLL) with disease progression on ibrutinib have worse outcomes compared to patients stopping ibrutinib due to toxicity. A better understanding of expected outcomes in these patients is necessary to establish a benchmark for evaluating novel agents currently available and in development. We evaluated outcomes of 144 patients with CLL treated at Mayo Clinic with 2018 iwCLL disease progression on ibrutinib. The median overall survival (OS) for the entire cohort was 25.5 months; it was 29.8 months and 8.3 months among patients with CLL progression (*n* = 104) and Richter transformation (*n* = 38), respectively. Longer OS was observed among patients with CLL progression who had received ibrutinib in the frontline compared to relapsed/refractory setting (not reached versus 28.5 months; *p* = 0.04), but was similar amongst patients treated with 1, 2, or ≥3 prior lines (18.5, 30.9, and 26.0 months, respectively, *p* = 0.24). Among patients with CLL disease progression on ibrutinib, OS was significantly longer when next-line treatment was chimeric antigen receptor T-cell therapy (median not reached) or venetoclax-based treatment (median 29.8 months) compared to other approved treatments, such as chemoimmunotherapy, phosphoinositide 3’-kinase inhibitors, and anti-CD20 monoclonal antibodies (9.1 months; *p* = 0.03). These findings suggest an unmet need for this growing patient population.

## Introduction

Ibrutinib has demonstrated long-term efficacy in relapsed/refractory (median progression-free survival [PFS] 44.1 months) [1] and frontline (5-year PFS estimate 70%) [2] patient populations with chronic lymphocytic leukemia (CLL), leading a therapeutic renaissance of targeted therapies capable of more frequent durable responses among even high-risk patient populations (4-year PFS among patients with *TP53* alterations 79%) [3]. Despite these excellent outcomes, the majority of patients eventually discontinue ibrutinib treatment; disease progression and toxicity being the most common reasons. Patients who stop ibrutinib for disease progression have worse PFS and overall survival (OS) compared to patients who stop ibrutinib because of toxicity [4–6]. Alternative classes of targeted agents (e.g., BCL2 antagonists, phosphoinositide 3-kinase inhibitors [PI3Ki], and next-generation anti-CD20 monoclonal antibodies) are now readily available in the clinic and have shown promise in the management of patients with relapsed CLL [7–9]. In addition, auspicious new drugs, including non-covalent Bruton tyrosine kinase inhibitors (BTKi) such as nemtabrutinib and pirtobrutinib, are in development with preliminary studies showing impressive efficacy in relapsed CLL after ibrutinib failure [10, 11]. Finally, cellular therapies (including CAR-T and allogeneic stem cell transplant) also represent important treatment options that need to be considered for this group of high-risk patients. A better understanding of expected clinical outcomes in patients with disease progression on ibrutinib is necessary to establish a benchmark for evaluating future studies related to the actual event and options for therapy. Here, we focus on outcomes after progression on ibrutinib in a large cohort of patients with CLL, reporting survival estimates with varied treatments, line-of-therapy settings, and patterns of progression.

## Methods

After IRB approval, we reviewed the medical records of patients with CLL who received ibrutinib therapy for CLL at a Mayo Clinic Cancer Center sites (Arizona, Florida, or Minnesota) between 4/2012–6/2021. Baseline relevant clinical characteristics, prior therapies, duration of ibrutinib treatment, and post-ibrutinib therapy were abstracted for all patients. Date of progression on ibrutinib therapy was ascertained by retrospective chart review and was documented according to the 2018 iwCLL guidelines [12]. Resistance mutation sequencing was conducted at NeoGenomics reference laboratory; methods are included in the Supplemental Materials.

Treatment-free survival (TFS) was analyzed as the duration from the start of treatment immediately after ibrutinib failure to the start of the subsequent line of therapy or death, whichever occurred earlier. Overall survival (OS) was analyzed as the time from date of progression while on ibrutinib and from subsequent therapy start date until date of death or last known to be alive. OS was analyzed using the Kaplan-Meier method (with comparisons of OS by characteristics analyzed by the log rank test) and Cox proportional hazards model. The association with OS and the event of venetoclax treatment at any time post-progression was analyzed as a time-dependent covariate in Cox proportional hazards models. Statistical analyses were conducted using SAS 9.4.

## Results

### Patients and disease characteristics

A total of 144 patients were identified who had progression of disease on ibrutinib therapy; 106 patients had progression of CLL, whereas 38 patients developed biopsy-proven Richter transformation (35 with diffuse large B-cell lymphoma [DLBCL], 3 with classical Hodgkin lymphoma). The characteristics of these 144 patients at the time of ibrutinib start as well as at the time of progression are shown in Table 1. The median age at the time of progression on ibrutinib was 68 years (range, 43–92). Ibrutinib was used as first-line therapy in 16% of patients. A total of 37/54 (69%) assessed patients had *BTK/PLCG2* mutations identified (19 *BTK* mutation only; 9 *PLCG2* mutation only; 9 both *BTK* and *PLCG2* mutations); 34/45 (76%) in patients with CLL progression and 3/9 (33%) in patients who experienced Richter transformation.

**Table 1:** Patient and disease characteristics at the time of ibrutinib start and time of progression on ibrutinib.

Parameter		Number (%) or Median [range]	
		At Ibrutinib Start	At Ibrutinib progression
Age, years		65 [40–91]	68 [43–92]
Males		85/115 (74%)	
Frontline therapy		23 (16%)	
Rai stage	0	24 (17%)	31 (22%)
	I–II	52 (37%)	40 (28%)
	III–IV	66 (46%)	72 (50%)
	Missing	2	1
Absolute Lymphocyte Count (x 10^9^/L)		24.3 [0.3–357.2]	7.6 [0.1–244.3]
	Missing	10	7
*IGHV* mutation status	Unmutated	100 (90%)	
	Missing	33	
FISH	None detected	18 (14%)	15 (13%)
	Other	2 (2%)	3 (3%)
	13q-	18 (14%)	20 (18%)
	Trisomy 12	13 (10%)	13 (11%)
	11q-	22 (18%)	15 (13%)
	17p-	53 (42%)	48 (42%)
	Missing	18	30
*TP53* Disruption (either del17p or *TP53* mutation)	Abnormal	57 (45%)	57 (50%)
	Missing	18	29
*BTK/PLCG2* mutation	Present	Not applicable	37 (69%)
	No mutation detected		17 (31%)
*BTK* mutation alone	Present		19 (35%)
*PLCG2* mutation alone	Present		9 (17%)
*BTK* and *PLCG2* mutations	Present		9 (17%)

### Survival outcomes after progression on ibrutinib among the overall cohort

**Fig. 1:**
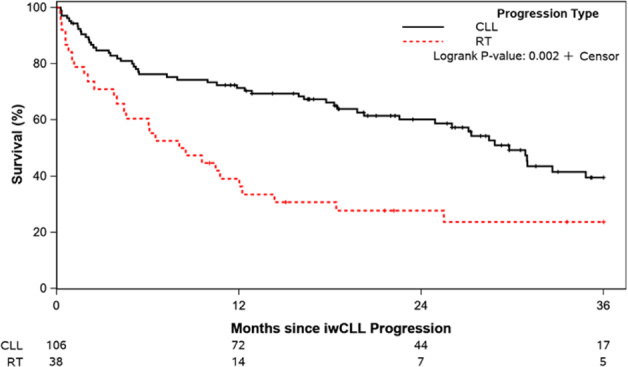
Overall survival from the time of progression event on ibrutinib treatment. Patients with CLL disease progression are compared to those who had Richter transformation events.

**Fig. 2:**
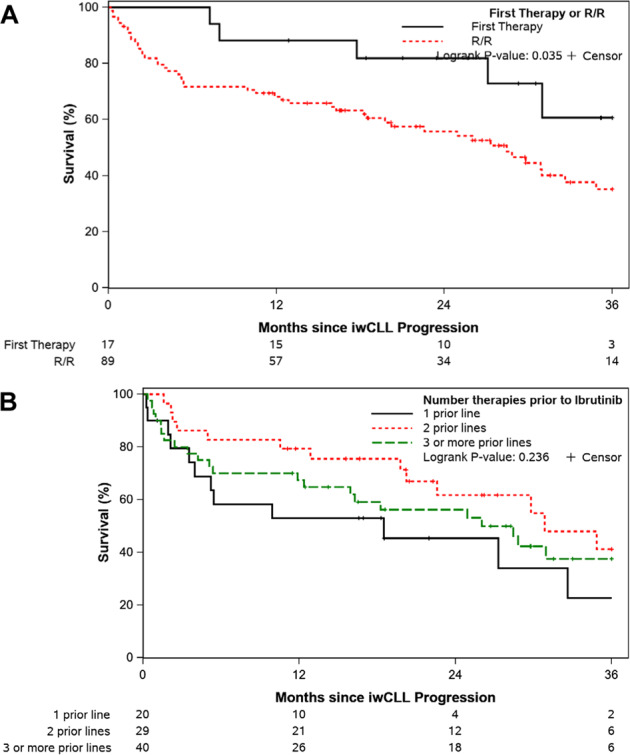
Overall survival from time of CLL progression on ibrutinib by line of therapy. **A** It compares patients who received ibrutinib ast first therapy to those treated with ibrutinib in the relapsed/refractory setting. **B** It further evaluates the relapsed/refractory cohort by number of prior lines of therapy.

**Fig. 3:**
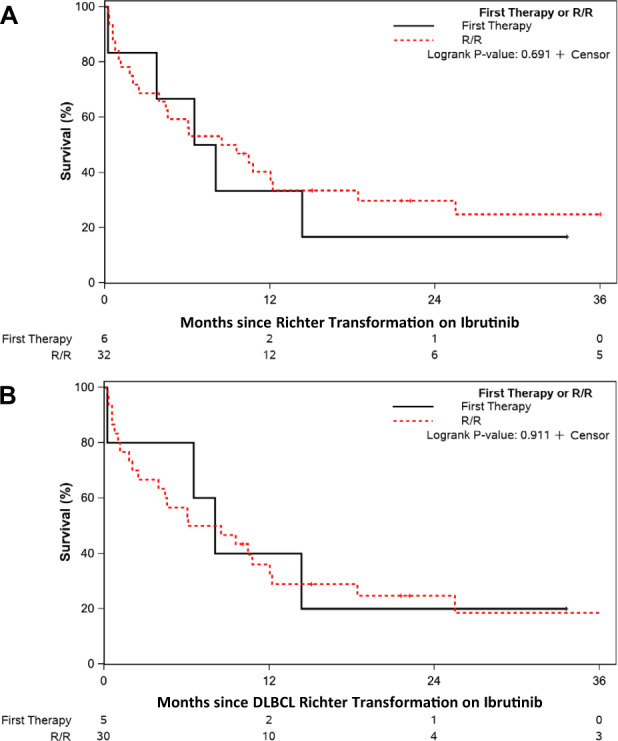
Overall survival following Richter transformation on ibrutinib by line of therapy. **A** It compares overall survival between all patients who experienced Richter transformation (diffuse large B-cell lymphoma or Hodgkin lymphoma) while receiving firstline ibrutinib to those in the relapsed/refractory setting. **B** It considers only patients with diffuse large B-cell lymphoma Richter transformation events.

The median OS of the entire cohort after progression on ibrutinib was 25.5 months (95% CI 17.7-31.0). Not unexpectedly, the OS was significantly different between those who experienced CLL disease progression versus Richter transformation (median 29.8 versus 8.3 months, respectively; *p* = 0.002, Fig. 1). The median OS of patients who experienced CLL disease progression when ibrutinib was used in the first-line setting was longer compared to those treated in the relapsed/refractory setting (not reached versus 28.5 months; *p* = 0.04; Fig. 2A). The median follow-up of patients from time of progression was 16.6 months overall; 23.5 months among patients treated in the first-line setting and 15.9 months in the relapsed/refractory setting. Among patients treated in the relapsed/refractory setting, the median OS when ibrutinib was used after one prior line (*n* = 20), 2 prior lines (*n* = 29), or ≥3 prior lines (*n* = 40) was 18.5, 30.9, and 26.0 months, respectively (*p* = 0.24; Fig. 2B). Similar rates of venetoclax-based treatment as immediate next-line therapy were used amongst these three groups (1 prior line, 2 prior lines, ≥3 prior lines): 60%, 45%, and 50%, respectively. The median OS of patients who experienced Richter transformation based on whether ibrutinib was used as first-line CLL therapy (*n* = 6) or in the relapsed/refractory setting (*n* = 32) was 7.3 versus 9.0 months (*p* = 0.69; Fig. 3A). Similarly, the median OS of patients who experienced diffuse large B-cell lymphoma (DLBCL) transformation was not significantly different whether ibrutinib was used as first-line therapy (*n* = 5) or in the relapsed/refractory setting (*n* = 30) (8.1 versus 7.3 months, respectively; *p* = 0.91; Fig. 3B).

### Clinical presentation and outcomes in patients with CLL progression

**Fig. 4:**
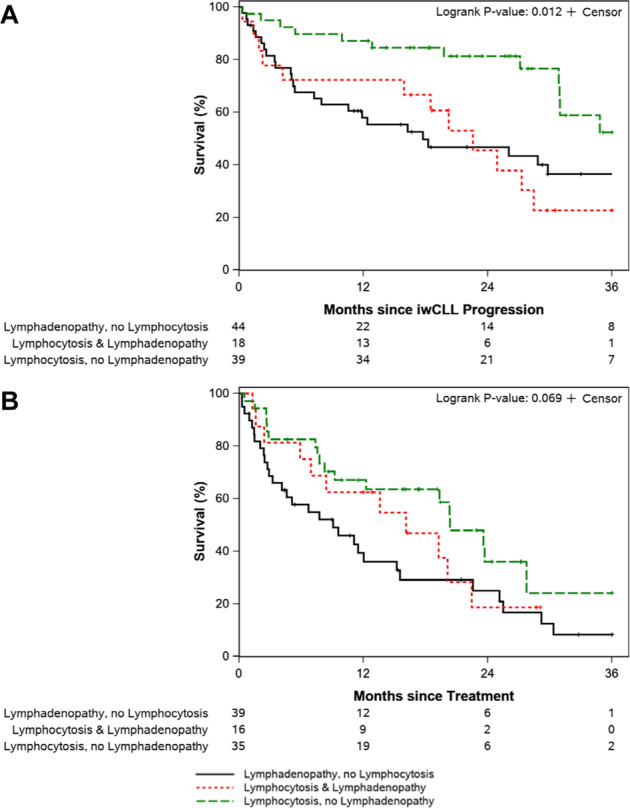
Overall survival and treatment-free survival among patients with CLL disease progression on ibrutinib by pattern of progression. **A** It compares overall survival by pattern of progression: lymphocytosis without lymphadenopathy, lymphadenopathy without lymphocytosis, or lymphocytosis and lymphadenopathy. **B** It compares treatment-free survival between the same groups. Note, the five patients with iwCLL-defined disease progression but without lymphadenopathy or lymphocytosis (i.e., biopsy-proven marrow infiltration causing cytopenias, progressive hepatosplenomegaly) are not included in this analysis.

Heterogenous patterns of progression were observed: progressive lymphadenopathy without concurrent lymphocytosis (*n* = 44); progressive lymphocytosis without concurrent lymphadenopathy (*n* = 39); concurrent progressive lymphadenopathy and lymphocytosis (*n* = 18). Progression without lymphadenopathy or lymphocytosis (i.e., biopsy-proven marrow infiltration causing cytopenias, progressive hepatosplenomegaly) was seen in only five patients. Presentation of progression was associated with OS with a trend towards a difference in TFS (Fig. 4A, B). The median OS from time of progression on ibrutinib was 17.7 months (95% CI, 10.5–40.6 months) among patients with lymphadenopathy without lymphocytosis, 22.6 months (95% CI, 15.9—not estimable) among patients with lymphadenopathy and lymphocytosis, and 46.7 months (95% CI, 31.0-not estimable) among patients with lymphocytosis without lymphadenopathy (*p* = 0.012). *TP53* disruption was not associated with pattern of progression (*p* = 0.86).

### Treatment outcomes in patients with CLL progressive disease

Among all patients with CLL progression (*n* = 106), the median time from iwCLL-defined progression to start of subsequent therapy was 1.4 months (95% CI, 1.0–1.8 months; range 0.1–23.5 months). The most common first salvage therapy consisted of a venetoclax-based regimen *n* = 56, PI3Ki-based treatment *n* = 11, chemoimmunotherapy *n* = 11, chimeric antigen receptor T-cell therapy (CAR T) *n* = 6, anti-CD20 monoclonal antibody treatment *n* = 4, and other therapies (comprised of various clinical trials) *n* = 6. Seven patients died before any salvage therapy could be administered because of progressive disease. Five patients continued ibrutinib therapy (±anti-CD20 monoclonal antibody) despite iwCLL progression for a median of 12.9 months (range 2.1–23.5 months).

**Fig. 5:**
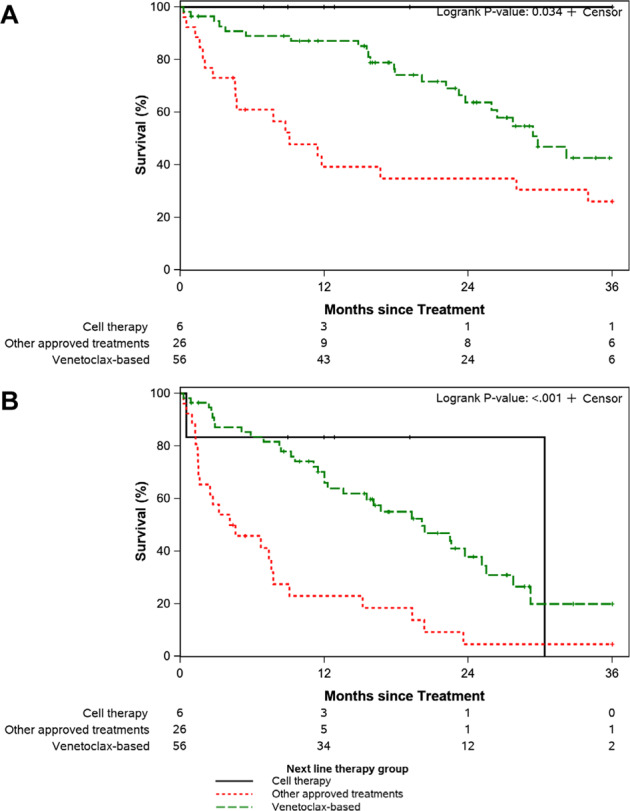
Survival outcomes following CLL progression on ibrutinib by subsequent line of therapy. **A** It compares overall survival between patients subsequently treated with CAR T-cell therapy, venetoclax-based treatment regimens, or other approved treatments. **B** It compares treatment-free survival between the same groups.

**Fig. 6:**
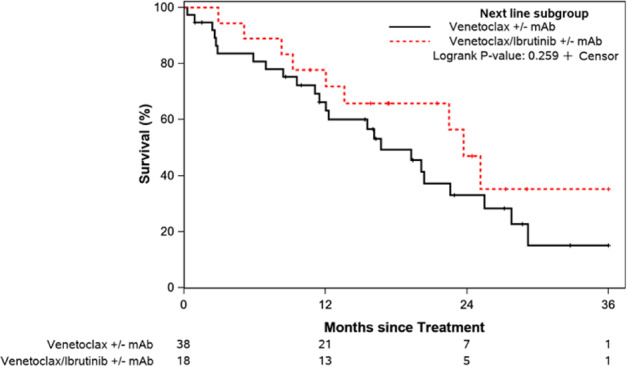
Treatment-free survival with venetoclax-based first subsequent therapy following CLL progression on ibrutinib. Outcomes of patients treated with continued ibrutinib as part of their venetoclax-based treatment are compared to those without continued ibrutinib.

The median OS was significantly longer among patients treated with CAR T or venetoclax-containing regimens compared to other approved treatments (e.g., chemoimmunotherapy, PI3Ki-based, and anti-CD20 monoclonal antibody) at not reached, 29.8 months, and 9.1 months, respectively (*p* = 0.034; Fig. 5A). The median TFS was also significantly longer in patients receiving CAR T or venetoclax-based regimens compared to other approved treatments at 30.4 months, 20.1 months, and 4.4 months, respectively (*p* < 0.001; Fig. 5B). Additional OS and TFS analyses of patients divided into smaller treatment subgroups are shown in the Supplemental Materials. Patients who started post-ibrutinib salvage treatment before April 2016 (approval date of venetoclax for relapsed CLL in the U.S.) had shorter OS (median 2.6 versus 31.0 months; *P* = 0.005). However, receipt of venetoclax treatment any time following progression on ibrutinib (e.g., not only as immediate next therapy) was not associated with better OS (HR 1.0, 95% CI 0.6–1.6; *p* = 0.89). Among the 56 patients who received venetoclax-based first subsequent therapy, 38 patients received venetoclax (±anti-CD20 monoclonal antibody) and 18 patients received venetoclax plus continued ibrutinib (±anti-CD20 monoclonal antibody). Patients who continued ibrutinib with venetoclax-based treatment had similar TFS to those who did not continue ibrutinib (median 23.7 versus 16.7 months; *p* = 0.26; Fig. 6). *TP53* disruption was not associated with TFS (HR 1.2, 95% CI 0.7–2.1; *p* = 0.51).

In univariate analyses, *IGHV* mutation status, *TP53* disruption, and the presence of a *BTK* or *PLCG2* mutation status were not predictors of shorter OS from time of CLL disease progression on ibrutinib. Time from iwCLL progression to start of subsequent therapy ≥1.5 months versus <1.5 months was associated with longer OS from subsequent therapy (47.1 versus 25.6 months; *p* = 0.03).

### Treatment outcomes in patients with Richter transformation

Among the 35 patients who had transformation to DLBCL, the most common first salvage therapy consisted of chemoimmunotherapy in 15 patients, immune checkpoint inhibitor therapy in nine patients, venetoclax-based therapy in three patients, and PI3Ki-based treatment, anti-CD20 monoclonal antibody treatment, and antibody drug conjugate treatment in two patients each. Two patients died before any salvage therapy could be administered because of progressive disease. The OS and TFS of these patients according to the types of treatments administered is shown in Supplemental Figs. 2A and 2B, respectively. Type of treatment did not have a significant impact on OS nor TFS.

Among the three patients who had transformation to Hodgkin lymphoma, two remained alive at last follow-up >4 years after receipt of ABVD (doxorubicin, bleomycin, vinblastine, dacarbazine) with no relapse of Hodgkin lymphoma. One patient died amidst neutropenic infection while in a partial remission after treatment with BCVPP (carmustine, vinblastine, cyclophosphamide, procarbazine, prednisone).

## Discussion

Here, we report on a meaningfully large cohort of nearly 150 patients showing a median overall survival of 25.5 months from the time of iwCLL progression on ibrutinib. This is strikingly similar to that reported in a recent abstract from The Ohio State University group on a similar cohort (median 24.4 months) [13]. Outcomes beyond CLL progression on ibrutinib were similar among those treated in the relapsed/refractory setting irrespective of number of prior lines, but differed by immediate subsequent therapy, favoring venetoclax-based and CAR T treatments. Key clinical observations at the time of progression, including pattern of progression and time from progression to next therapy start, also showed prognostic OS relevance in our study. These data improve our current understanding of outcomes in this growing patient population and provide a critical benchmark when considering trials of promising novel agents in development. Treatment patterns and outcomes following disease progression on a BTKi among CLL patients are less well represented in the randomized, prospective clinical trials of approved treatments in the relapsed/refractory space [7, 8]. Extrapolating predictions for patients with ibrutinib-refractory disease from data in ibrutinib-exposed patients is problematic and therapeutic decisions are guided by single-arm prospective studies, subgroup analyses, and limited retrospective cohorts.

Previous studies have shown a limited benefit to be expected with PI3Ki or chemoimmunotherapy (median PFS 9 months and 5.1 months, respectively) in patients who previously received ibrutinib and stopped for any reason [14]. Our study demonstrates patients with CLL disease progression on ibrutinib have better survival outcomes with venetoclax-based regimens compared to other approved options (median OS 29.8 versus 9.1 months; median TFS 20.1 versus 4.4 months). In the prospective study of venetoclax monotherapy post-BTKi treatment, the median PFS was 24.7 months and 12-month OS rate 91%, without separating that cohort by exposed and refractory subgroups. Overall response was higher among patients non-refractory to prior BTKi treatment (63% versus 54%) [15]. A pooled analysis of these patients and others treated with venetoclax on a clinical trial post-BTKi reported an ORR of ~65% without differentiating BTKi-exposed versus –refractory [16]. In our cohort, venetoclax at any time post-ibrutinib progression did not impart the same OS benefit observed when analyzing next-line venetoclax compared to alternative options. While difficult to reconcile these findings entirely, comparing outcomes pre- and post-venetoclax approval date also hints at an importance in sequence of therapies. Altogether, our new results and these previously reported data support the most common current practice of proceeding to venetoclax-based treatment as next-line treatment following progression on ibrutinib in venetoclax-naïve patients. CAR T also has therapeutic efficacy in highly refractory patients but lacks current approval [17]. Patients treated with CAR T in our cohort had outstanding outcomes (illustrated by a median OS not reached with no events among the six patients), but conclusions are precluded by the limited number of patients and the optimal timing to pursue cellular therapy in the novel agent era remains unknown.

Considering the risk for disease flare with ibrutinib interruption, particularly amidst progressive disease, the synergistic combination of ibrutinib and venetoclax has appeal in the post-ibrutinib progression setting as well [18]. However, when focusing on the venetoclax-based treatment subgroups in this study, no significant difference in TFS was observed with continued ibrutinib. Results have not yet been reported for a prospective trial (NCT03422393) evaluating dose-escalated ibrutinib and the addition of venetoclax for next-line treatment at ibrutinib progression.

We demonstrated the pattern of progression, specifically progressive lymphadenopathy, was associated with post-progression OS. Median survival estimates among the pattern of progression subgroups (17.7 months and 46.7 months for patients with lymphadenopathy without lymphocytosis and lymphocytosis without lymphadenopathy, respectively) closely resembled those presented by the OSU group (15.2 months and 49.9 months for the same groups) [13]. Better understanding this difference is an area of active research. A possible contribution could be differing mechanisms of resistance. Resistance mutations in *BTK* and *PLGC2* were less frequently detected among patients relapsing with lymphadenopathy (40%) compared to those with lymphocytosis (81%) in a prior study [19]. Another potential reflection of varied CLL biology at relapse on ibrutinib is our finding that patients who received subsequent treatment ≥1.5 months beyond relapse had OS that was approximately twice as long as patients receiving next-line therapy sooner. One possible reason for this apparent paradox is that the patients progressing in a more gradual, less dramatic fashion and thus in less need of urgent change in therapy are biased to more favorable outcomes, similar to the diagnosis-to-treatment interval shown in patients with newly diagnosed large cell lymphoma [20].

Survival following Richter transformation to DLBCL on ibrutinib was similarly dismal whether occurring in the frontline or relapsed/refractory setting, emphasizing the continuing urgent need for better Richter transformation treatments. Outcomes differed between frontline and relapsed settings in those with CLL progression with longer survival observed following progression on frontline ibrutinib. Unexpectedly, among patients who received ibrutinib in the relapsed setting, post-progression survival was similar across patients with one to three or more prior lines of therapy. This finding seems to place emphasis on treatment after progression on ibrutinib but requires validation in independent cohorts.

The majority of ibrutinib treatment in this study occurred in the relapsed/refractory setting, which may be less reflective of contemporary practice and is a limitation of the study. Our experience here is that of a tertiary referral center, which likely explains the higher-than-expected number of Richter transformation cases observed and should not be interpreted as a true incidence rate. Exploring the impact of resistance mutations in outcomes and patterns of progression is limited due to lack of consistent sequencing in this cohort. Similarly, the limitation of non-uniform follow-up inherent to a retrospective study precludes reporting a reliable response assessment. However, strengths include (1) our focus on meaningful TFS and OS outcomes; (2) description of clinical features of the disease progression while on ibrutinib made possible by a well-annotated, prospectively maintained CLL Database; (3) relatively long follow-up compared to earlier studies evaluating this patient population. These aspects of the study facilitate providing greater insight overall into outcomes of CLL patients in the post-ibrutinib progression period.

Results from this study demonstrate that progression of disease on ibrutinib represents an ongoing unmet need in patients with CLL. Although non-covalent BTKi, such as nemtabrutinib and pirtobrutinib, show promising efficacy in this setting, they currently lack label approval and are only available through a clinical trial [10, 11]. Participation in well-designed clinical trials is remains key for this growing patient population. Venetoclax-based treatments (if not given before) are the current standard in the clinic for this group and indeed offered amongst the best TFS and OS outcomes in this study. We speculate that the role for continued ibrutinib beyond progression in combination with venetoclax in certain patients and the impact and optimal timing of cellular therapy remain important questions.

## Supplementary information

Supplemental Materials

## Data Availability

Data not available without request and IRB review due to patient confidentiality.
