# Supplementary material for: Clinical outcomes in patients with chronic lymphocytic leukemia with disease progression on ibrutinib
Source: Blood Cancer J. 2022 Sep 1;12(9):124. doi: 10.1038/s41408-022-00721-6 (PMC9437078; doi:10.1038/s41408-022-00721-6)
Supplement: Supplementary file 1 — Supplemental Materials [file 41408_2022_721_MOESM1_ESM.pdf]

Supplemental Figure 1:

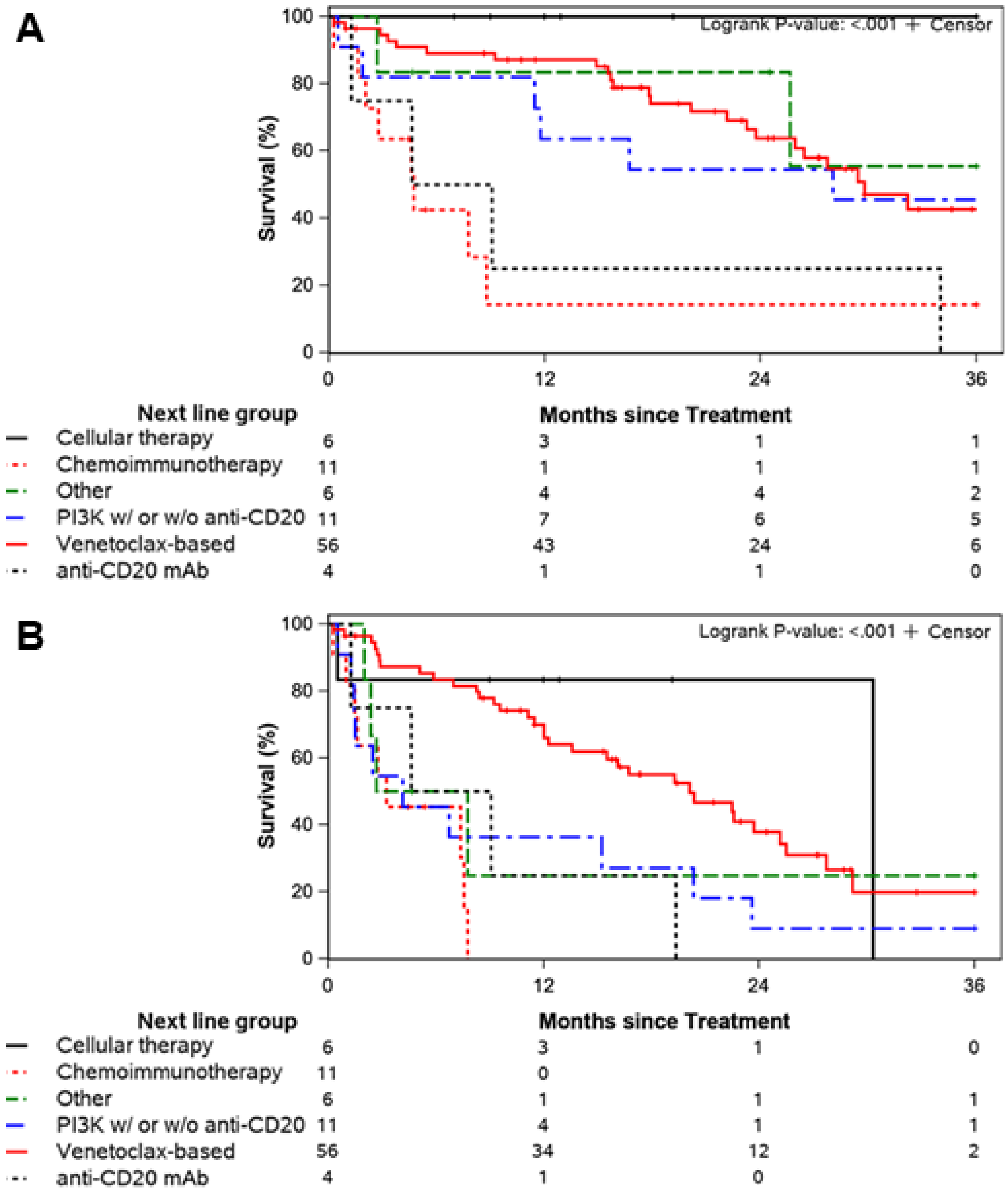

Caption: **(A)** Overall survival and **(B)** treatment-free survival following CLL progression on ibrutinib by subsequent line of therapy group.

Supplemental Figure 2:

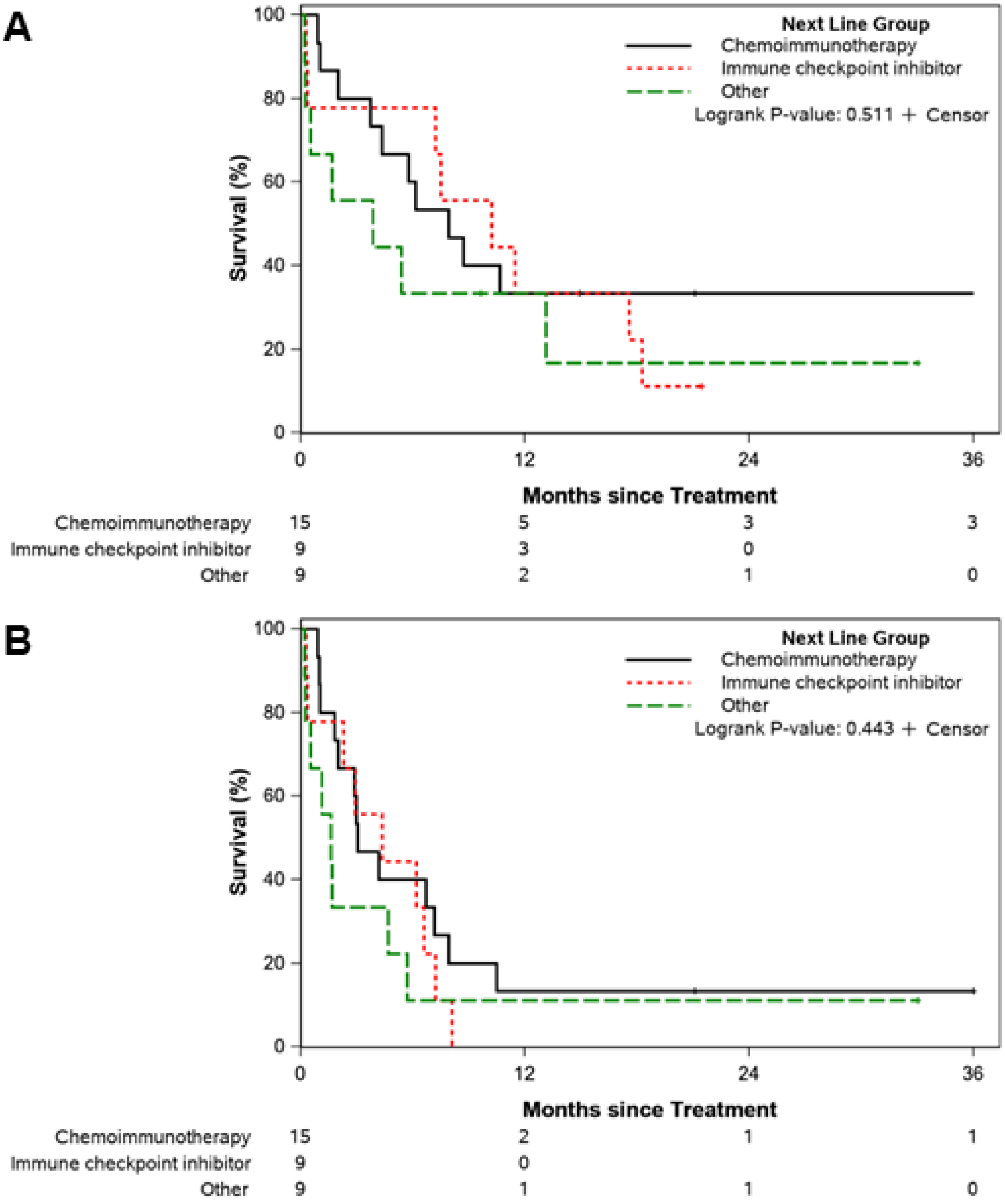

Caption: **(A)** Overall survival and **(B)** treatment-free survival following Richter transformation to 433 diffuse large B cell lymphoma on ibrutinib by subsequent line of therapy group.

Supplemental Methods

Mutation analysis of *BTK* and *PLCG2* was performed commercially by NeoGenomics Laboratories. In brief, nuclei acid was isolated from peripheral blood cells, followed by high-sensitivity Sanger sequencing of mutation hotspot regions of exon 15 of the *BTK* gene, (including C481S mutation to 0.1% mutant allele in a background of wildtype), and exons 19, 20, and 24 of *PLCG2* gene which includes R665W, S707Y, and L845F mutations to approximately 0.16% to 1.0%.
